# The concept of an agroinfiltration kit for recombinant protein production for educational and commercial use—A journey through a forest of regulatory and legal implications

**DOI:** 10.3389/fbioe.2022.926239

**Published:** 2022-09-05

**Authors:** Holger Spiegel, Greta Nölke, Harry Thangaraj, Stefan Schillberg

**Affiliations:** ^1^ Fraunhofer Institute for Molecular Biology and Applied Ecology IME, Aachen, Germany; ^2^ Independent Consultant, (Residential Address Withheld), Finchley, London, United Kingdom; ^3^ Justus-Liebig-Universität Giessen, Department of Phytopathology, Giessen, Germany

**Keywords:** *Agrobacterium tumefaciens*, Nagoja protocol, *Nicotiana benthamiana*, recombinant protein expression, transient transformation

## Abstract

Recombinant expression using Agrobacterium-mediated transient transformation (ATT) of plants has developed into a robust and versatile method to rapidly produce proteins. The capability of plants to efficiently synthesize even homo- and hetero-multimeric complex folded proteins featuring disulfide bonds and other post-translational modifications such as N-linked glycosylation makes them superior to most of the established microbial, especially prokaryotic expression hosts. Compared to production in mammalian cell cultures, ATT requires lower skills, simple technical equipment and cheaper media components. Taken together these features make the method optimally suited for R&D applications involving the development and engineering of recombinant proteins for various purposes ranging from vaccine candidates, therapeutic proteins, towards enzymes for different pharmaceutical and technical applications. Despite these advantages the technology is currently not being used outside the community of plant research. The design and realization of a kit containing all the information, instructions and ideally also the material required to perform recombinant protein production using ATT in an educational or commercial context was one of the objectives of the EU-funded Horizon 2020 project Pharma-Factory. While it is pretty straightforward to assemble a comprehensive instruction manual describing the procedure, the clarification of regulatory and legal aspects associated with the provision, dissemination and use of the different materials and organisms required to perform ATT is a complex matter. In this article, we describe the initial concept of an ATT kit for educational as well as research and development (R&D) purposes and the specific regulatory and legal implications associated with the various kit components. We cover aspects including intellectual property rights, freedom-to-operate (FTO), safety regulations for distributing genetically-modified organisms (GMOs), as well as export and import regulations. Our analysis reveals that important components of the ATT kit are freely available for research purposes but not or only with considerable effort for commercial use and distribution. We conclude with a number of considerations and requirements that need to be met in order to successfully disseminate such a kit in the future.

## 1 Introduction

The technology of Agrobacterium-mediated transient transformation (ATT) of plants and plant tissue is based on a natural gene-transfer mechanism (T-DNA transfer) found in the soil organism *Agrobacterium tumefaciens*. Wild-type agrobacteria use this mechanism to hijack infected host plant’s metabolism and utilize it for the production of tumor-inducing compounds as well as special secondary metabolites called opines that serve as carbon and nitrogen source for the pathogen (Vladimirov et al., 2015). Not long after its discovery in the mid-seventies plant scientists began to utilize the mechanism and the responsible genetic elements to enable efficient delivery of transgenes into plant cells (see Zupan et al., 2000 for review). In wild-type agrobacteria these elements are located on the tumor inducing (Ti) plasmid. Engineered *A. tumefaciens* strains used in plant biotechnology for ATT carry disarmed Ti-Plasmids lacking tumor inducing ability while still encoding the functions required to mediate T-DNA transfer into infected plant cells (Zambryski et al., 1983). A so called binary vector is used to facilitate the transfer of a gene of interest (GOI) into the plant genome (Bevan, 1984). This plasmid replicates in *Escherichia coli* (for cloning purposes) and *A. tumefaciens* (to perform ATT) and contains selectable markers for both organisms. The core element of the binary vector is an expression cassette for a protein of interest (POI), located between the flanking sequences (left (LB) and right border (RB)) to mediate T-DNA transfer (Zupan and Zambryski, 1995; Stanton, 2003). After transformation of a binary vector into a suitable *A. tumefaciens* strain (Hellens et al., 2000) the bacteria can be cultivated and subsequently used to transform plants stably (Hamilton et al., 1996) or plants and plant parts transiently by infiltrating agrobacterium suspension into the tissue either by manual syringe infiltration or by vacuum infiltration (Krenek et al., 2015; Spiegel et al., 2022). After an incubation (including lighting and watering/humidification) period of three to 6 days, plants can be harvested for recombinant protein extraction and purification. Because of its simplicity and the possibility to separately infiltrate single leaves of one plant, the method is excellently suited for protein engineering, when different variants of proteins need to be rapidly produced and tested at a small scale, e.g. in the context of vaccine candidate development or for the evaluation of diagnostic reagents (Vaquero et al., 1999; Boes et al., 2016; Pietschmann et al., 2021).

Besides being fast and robust, the method provides unmatched flexibility through the possibility to perform so-called co-infiltration with several recombinant agrobacterium cultures harboring binary vectors that encode different proteins. Using this strategy it is possible to facilitate the transient expression of complex multi-domain proteins like immunoglobulins (Vézina et al., 2009) composed of up to four different protein chains (e.g. IgA featuring a heavy chain, a light chain, a joining chain, and a secretory component). Using adjusted quantities of agrobacterium cultures, it is also possible to successfully optimize the recombinant production of multimeric proteins with biased stoichiometry. Selected genetic elements like promotors, untranslated regions (UTRs) as well as various targeting signals within the gene expression cassette can be utilized to adjust expression levels, and subcellular localization of the recombinant POIs, which adds another level of flexibility. Furthermore binary vectors can be set-up in a way to accept multiple gene expression cassettes allowing the convenient introduction of complex pathways and subsequent testing using ATT (Goderis et al., 2002). Additionally, it is possible to cryopreserve recombinant agrobacteria in the form of “ready to use stocks” that can be instantly used for ATT experiments (Spiegel et al., 2019).

One of the goals of the EU Horizon 2020 project Pharma-Factory (https://pharmafactory.org) is the design and dissemination of a kit enabling molecular biologists, who have not previously worked with this technology, to produce recombinant proteins using ATT. The concept of “easy to use” kits that lower the entry barrier for novel technologies is not completely new, and recent examples are educational kits that have been designed to provide an insight into the functionality of genome editing tools using the CRISPR/Cas9 or the CRISPR/Cas12 system. Several companies offer kits aimed specifically at students, including Bio-Rad (Hercules Ca, United States), Rockland (Limerick, Pa, United States) as well as organizations affiliated with universities like Innovative Genomic Institute (Berkley, Ca, United States). These kits enable the CRISPR-based knockout of genes whose expression can be followed via visual markers (beta-galactosidase) and usually contain lyophilized bacteria (*E. coli*), plasmid DNA, antibiotics and media components, along with comprehensive tutorials. These kits can be used in schools and universities for educational purposes and have been optimized to function reliable in various environments with minimal technical infrastructure and basic microbiology/molecular biology skills. In the following we describe our considerations and conclusions regarding ATT kit concepts and the associated legal and regulatory implications.

## 2 Concept and legal implications

### 2.1 Considerations for the kit concept

**FIGURE 1:**
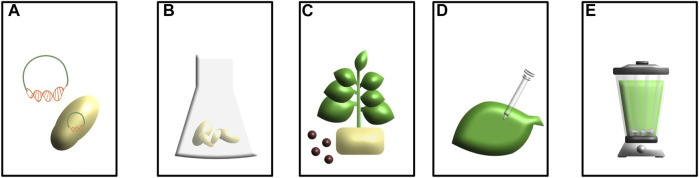
Overview of the workflow of *A. tumefaciens*-mediated transient expression in plants. **(A)** Cloning and transformation, **(B)** Cultivation of *A. tumefaciens*, **(C)** Cultivation of *N. benthamiana* plants, **(D)** Syringe infiltration and incubation of plant material, **(E)** Protein extraction and purification.

During the design phase different scenarios for the composition of an agroinfiltration kit were tested for feasibility. The initial idea was to assemble a collection of materials and instructions that would enable the recipient to produce recombinant proteins in plants using ATT without the need for further acquisition of components other than standard lab reagents. To define essential components and process steps of ATT a comprehensive visual representation of the process and its components was generated as basis for further considerations (Figure 1). To characterize the process in sufficient details it was broken down into the following sections: cloning and transformation of *A. tumefaciens*, cultivation of recombinant agrobacteria, cultivation of *Nicotiana benthamiana* plants, syringe infiltration and cultivation of intact plants or leaves, as well as protein extraction and purification. Based on further analysis and compilation of required material, equipment, and skills it became apparent that depending on available skills and infrastructure two different user groups (scientific and educational) can be defined and should be addressed by two different kit concepts, one kit for R&D applications and a second for the educational sector.

#### 2.1.1 R&D ATT kit

**FIGURE 2:**
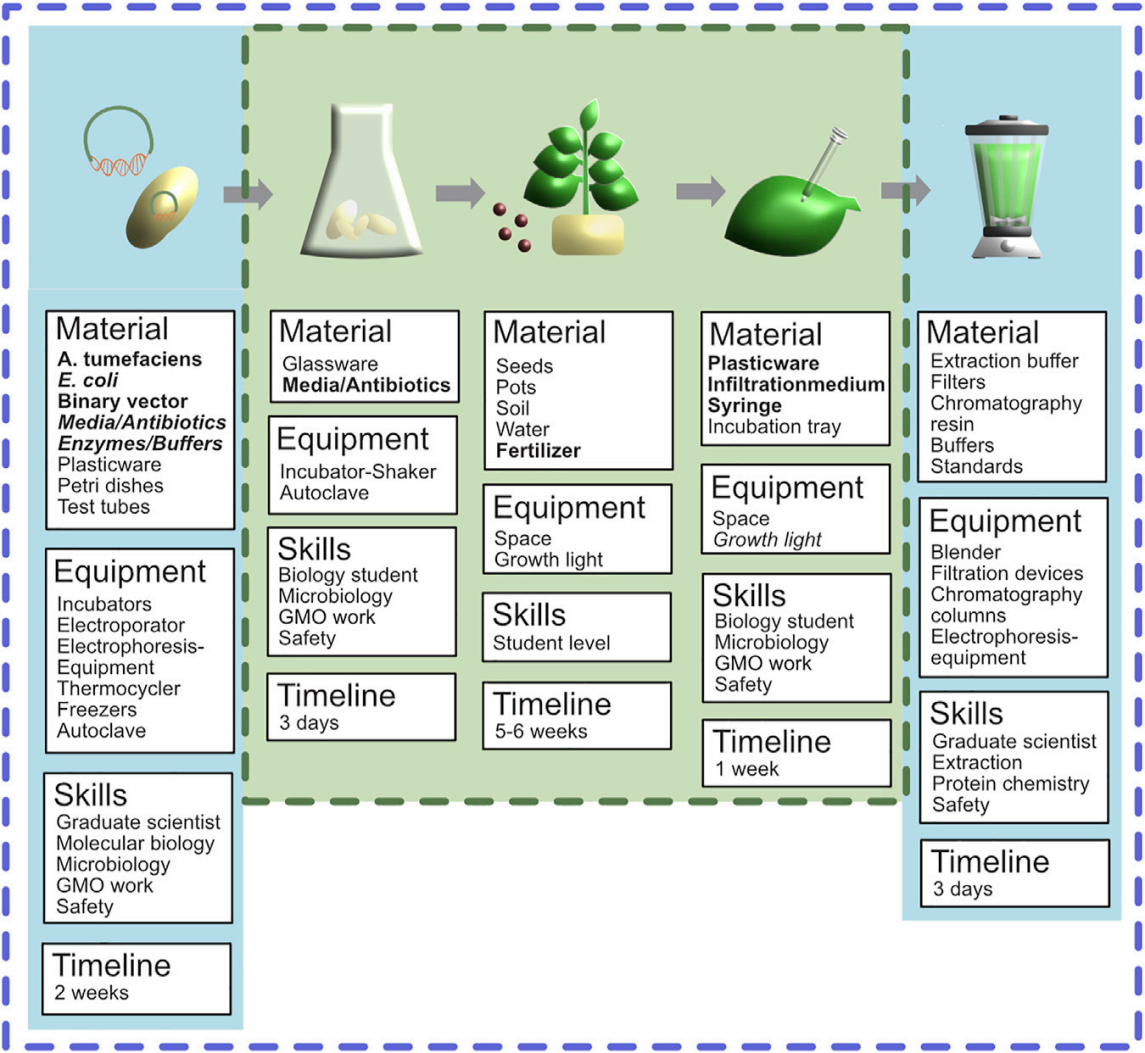
Illustration and listing of components associated with the different steps of ATT. Dark blue dashed line covers the procedure addressed by the R&D ATT kit. Dark green dashed line covers the core part of the procedure addressed by the educational ATT kit.

The R&D kit should enable experienced molecular biologists in an established molecular biology laboratory environment to perform the expression of recombinant proteins using ATT. To ensure maximal flexibility and to keep the kit as compact as possible, the R&D version should contain only the minimal components required to perform the procedure. Therefore, the R&D ATT kit should contain the following components: (i) a binary vector featuring a multiple cloning site for the flexible integration of genes encoding the POI, (ii) a disarmed *A. tumefaciens* strain compatible with the binary vector, and (iii) *N. benthamiana* seeds. In addition, the kit should be equipped with (iv) a manual that provides vector maps, general considerations, reagent and material lists as well as all protocols and a link to a video tutorial, which is useful for performing ATT. Materials, equipment, skills and timelines required to perform ATT based on the R&D kit are summarized in Figure 2, which also covers the respective information for the educational ATT kit.

#### 2.1.2 Educational ATT kit

The educational ATT kit concept addresses educational institutions and environments with limited cabilities to perform molecular cloning work due to the lack of equipment (e.g. centrifuges, gel-electrophoresis units) and experience. The idea of this kit is to disseminate the awareness that plants can be used as alternative production system for recombinant proteins, and that ATT represents a flexible, robust and fast alternative to established microbial and mammalian expression systems. The designated user group here are educational institutions like schools, universities or entities that provide advanced training for teachers in the field of biology and life sciences. To circumvent the need for any cloning work, the kit should feature: (i) a recombinant *A. tumefaciens* clone carrying a binary vector that codes for a marker protein that can easily be visualized by fluorescence with an included 488 nm laser pointer and a 500 nm filter foil, (ii) *N. benthamiana* seeds, and (iii) a selection of essential chemicals and consumables that are required for ATT. Further, (iv) the above mentioned ATT manual will also be a part of the educational ATT kit.

As shown in Figure 2, the successful implementation of ATT in either scenario (R&D and educational) requires certain equipment and laboratory infrastructure, as well as safety measures and country dependent regulatory approvals for GMO work. It is clear that in the R&D scenario the equipment requirements are not a major hurdle that would prevent the successful kit-based dissemination of the ATT technology since no “unusual” equipment is required. All hardware needed, including centrifuges, pipettes, incubators, etc. are usually available in a standard molecular biology laboratory, and can even be found in high schools with a focus on life sciences. Looking at the process workflow and the required material (summarized in Figure 2) it becomes obvious that especially the first and last process sections (cloning and protein purification) do require more specialized and expensive equipment (centrifuges, PCR cyclers, electroporators, filtration devices, electrophoresis and chromatography equipment) than the central steps (cultivation, infiltration incubation), and this will certainly prevent the performance of the whole procedure for educational purposes in schools. A second issue that affects all process steps is the fact that basically all ATT procedures involve the work with GMOs and require specific infrastructural features and regulatory approvals. Additionally when thinking about distributing microorganisms, plasmids, plant seeds as well as chemicals, various regulations ranging from intellectual property (IP) issues towards customs regulations and safety issues have to be considered. All the different regulations, requirements and their implications on the successful dissemination of ATT kits are further discussed in the following sections.

### 2.2 Legal implications associated with kit components

Examing the legal implications for all potential kit components and how they would affect or even restrict the kit’s composition is an essential task in the concept phase. In the following we have listed and described all the components of the kit: (i) the binary expression vector, (ii) *A. tumefaciens*, (iii) *N. benthamiana* seeds, and (iv) oligonucleotides and chemicals that are subject to legal implications and how these can be addressed.

#### 2.2.1 Binary expression vector

**FIGURE 3:**
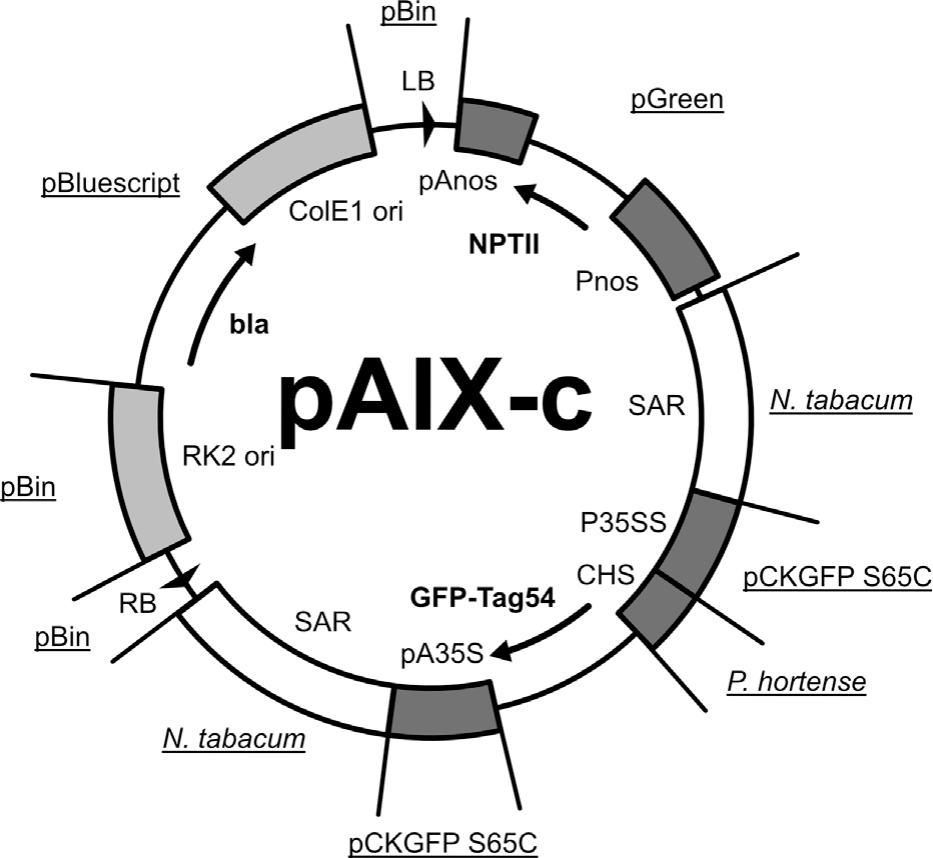
Illustration of the pAIX-c binary vector and its features. LB: left border of *A. tumefaciens* T-DNA; RB: right border of *A. tumefaciens* T-DNA; pAnos: polyadenylation sequence of the nopaline synthase of *A. tumefaciens*, Pnos: promotor of the nopaline synthase of *A. tumefaciens*; SAR: scaffold attachment region of the RB7 gene from *N. tabaccum*; P35SS: Promotor from *Cauliflower mosaic virus*; CHS: 5′ untranslated region of the chalcone synthase gene from *P. hortense*; pA35S: polyadenylation signal from *Cauliflower mosaic virus*; ColE1 ori: Origin of replication from ColE1 plasmid (*E. coli* compatible), RK2 ori: Origin of replication from RK2 plasmid (*A. tumefaciens* compatible); NPTII: kanamycin resistance gene; GFP-Tag54: Green fluorescent gene from *Aquorea victoria* with C-terminal 54k epitope tag sequence derived from *Tobacco mosaic virus*; bla: beta-lactamase gene from *E. coli*. Sources (plasmids and organisms) are underlined (see Table 1).

One of the main elements of the ATT technology is the binary expression vector containing the expression cassette for the gene of interest (GOI) flanked by regulatory elements that enable the Agrobacterium-mediated gene transfer into the plant chromosomes. With the aim of generating a binary vector for an ATT kit whose use is not restricted by any IP rights, we analyzed the IP situation of all genetic elements required for an ATT-compatible binary vector (Table 1) and designed the vector pAIX-c, which was completely synthetically produced. To our best knowledge, pAIX-c provides freedom-to-operate (FTO) regarding all the genetic elements used for its construction. A more detailed description of the FTO analysis for pAIX-c is has been published by Thangaraj (2022). The binary vector pAIX-c is a derivative of the binary vector pTRAc previously described (Maclean et al., 2007). A graphical representation of the pAIX-c vector is shown in Figure 3.

**TABLE 1:** Summary and analysis of pAIX-c genetic elements. N/A: Not applicable.

Elements	Description	Source	Patent(s)	FTO
LB	Left border region of T-DNA from *A. tumefaciens* nopaline Ti-plasmid pTiT37	pBinHygTOp, Genbank ID: Z37515	Expired	**✓**
RB	Right border region of T-DNA from *A. tumefaciens* nopaline Ti-plasmid pTiT37	pBinHygTOp, Genbank ID: Z37515	Expired	**✓**
Pnos	Nopaline synthase gene promoter	pGreen nos cassettes, www.pgreen.ac.uk	N/A	**✓**
NPT-II	Aph3′II (nptII): kanamycin resistance	pGreen nos cassettes, www.pgreen.ac.uk	N/A	**✓**
pAnos	Nopaline synthase gene polyadenylation signal	pGreen nos cassettes, www.pgreen.ac.uk	N/A	**✓**
SAR	Scaffold attachment region of the tobacco RB7 gene	*N. tabacum*	Expired	**✓**
P35SS	CaMV 35S promoter with duplicated transcriptional enhancer	pCKGFP S65C, MPI Cologne, Germany	Expired	**✓**
CHS 5′UT	5′ untranslated region from chalcone synthetase gene	*Petroselinum hortense*	N/A	**✓**
TL	5′ untranslated region from *Tobacco etch virus*	*Tobacco etch virus*	Expired	**✓**
GFP	Green fluorescent protein	*Aequorea victoria*	Expired	**✓**
Tag54k	Epitope recognized by mAb54k	54k protein from *Tobacco mosaic virus*, Rasche et al., 2011	Expired	**✓**
pA35SS	CaMV 35S polyadenylation signal	pCKGFP S65C, MPI Cologne, Germany	Expired	**✓**
RK2 ori	origin of replication	pBinHygTOp, Genbank ID: Z37515	N/A	**✓**
*Bla*	β-lactamase gene	pBluescriptII KS(-),Stratagene, La Jolla, CA, United States	None	**✓**
ColE1 ori	Origin of replication	pBluescriptII KS(-),Stratagene, La Jolla, CA, United States	None	**✓**

#### 2.2.2 *Agrobacterium tumefaciens*

To successfully perform ATT, the *A. tumefaciens* strain and the binary vector used in the procedure must be compatible regarding genetic elements (modified Ti helper plasmid) and antibiotic selection markers used to allow maintenance of the binary vector and Ti helper plasmid (Hamilton et al., 1996). Since it is widely used in research labs and compatible with the pAIX-c vector, we have chosen the *A. tumefaciens* strain GV3101:pMP90RK [Gentamicin, Kanamycin and Rifampicin resistance (GmR, KmR, RifR)] (Koncz and Schell, 1986) for the ATT kit concept. The strain can be obtained e.g. by the DSMZ, the German Collection of Microorganisms and Cell Cultures GmbH (https://www.dsmz.de) under the entry DSM 12365. There is no active IP protection associated to that strain but according to the documentation and after negotiations with the DSMZ commercial use and transfer to third parties is completely excluded for this strain. To our knowledge, there are no alternative sources of the *A. tumefaciens* strain GV3101 that can be used for commercial distribution of an ATT kit. The implications of this limitation are discussed below.

#### 2.2.3 *Nicotiana benthamiana* seeds

The preferred plant species to perform ATT is *N. benthamiana*, a solanaceous plant and wild relative of tobacco (*Nicotiana tabacum*) originally isolated from arid areas in Australia. It is widely used within many research labs throughout the molecular farming community which uses plants for production of valuable recombinant proteins (Schillberg and Finnern, 2021). It is a compact plant that only requires 50–70 cm of vertical space during cultivation and can be easily germinated and grown in greenhouses, phytochambers or even in laboratory space. Despite its widespread use, the origin of the seeds circulating within the scientific community is unknown although various efforts have been made to find appropriate documentation (Bally et al., 2018). It is known that most *N. benthamiana* seeds used in North American and European laboratories carry the Rdr1 insertion genotype making it more susceptible to various plant viruses. In most labs there is no proper certificate of origin for the *N. benthamiana* seeds that have been used for more than 2 decades. To avoid complications with German and European legislation regarding distribution of plants seeds (for more details refer to Section 3) we are still searching for a commercial source of *N. benthamiana* seeds providing proper documentation and grants commercial use and transfer to third parties.

#### 2.2.4 Oligonucleotides and chemicals

In the initial concept phase we also evaluated the possibility to include essential components like specific oligonucleotides (for colony PCR to verify the presence of the expression vector in recombinant agrobacteria) and certain ATT-specific chemicals (e.g. fertilizer for plant cultivation, 3,5-dimethoxy-4-hydroxyacetophenon for agrobacteria culture medium) into the kits to improve the convenience for the user. However, this idea was dropped after consideration of the various legal implications involved. Although it is possible to redistribute products purchased on the market, there are some aspects that need to be taken into account when doing so. Oligonucleotides and synthetic genes usually come with certain “limited label use” (LLU) licenses that restrict the use of the products in certain ways. Depending on the supplier sometimes these regulations restrict certain types of use (not for use in humans) and sometimes they prohibit distribution to third parties. When chemicals are distributed or transferred, there are usually several rules relating to safety and customs declaration that complicate shipping documentation (also refer to Section 3). Therefore, it is recommended to reduce the number of kit components to a minimum and to provide detailed information on the required oligonucleotides and chemicals in the manual to be ordered individually by the kit user.

### 2.3 Legal implications associated with the procedures and handling of GMOs

ATT procedures such as cloning, transformation and cultivation of agrobacteria, agroinfiltration as well as recombinant protein extraction and purification are long-established standard methods that can be considered “in the public domain” and are therefore no longer protected by IP. A basic FTO analysis is presented in Table 2.

**TABLE 2:** FTO analysis on methods involved in the ATT technology.

Methods	Consideration	FTO
Heat shock transformation of E. coli	Basic method described by Hanahan, 1983. Technology has not been patented	✓
Electro shock transformation of *A. tumefaciens*	Method described by McCormac et al., 1998. No active patent	✓
Preparation of plasmid DNA	Several basic methods have been described, e.g. by Sambrook et al., 1989, and patents are expired	✓
Agroinfiltration	Basic method by Kapila et al., 1997 has not been patented. Syringe infiltration of monocots may be IP protected through a claim on monocot transient transformation (US18602509P), but does not affect agroinfiltration of *N. benthamiana* plants	✓

Basically any technology (except *in vitro* transcription and translation) for production of recombinant proteins is based on the genetic manipulation of organisms. When performing the entire process of ATT, genetically modified *E. coli* will be generated during the cloning of the binary expression vector and genetically modified *A. tumefaciens* carrying this vector will be used for the transient expression procedure. This means that working with genetically modified organisms (GMO) cannot be avoided during ATT. Cloning strains of *E. coli*, and disarmed *A. tumefaciens* strains are generally classified as biosafety level 1 (BSL1, or Class1 according to the EC directive on GMO microorganisms (Directive 2009/41/EC)). There are different national and even subnational regulation that provide the legal framework for GMO work. The safety level classification is not only dependent on the GMO but can also be influenced by the GOI that is being introduced into the GMO. Introducing plasmids encoding toxic proteins may lead to an S2 classification for an organism normally being rated S1. In many countries, entities working with GMOs have to apply for the clearance to run a genetic engineering facility. According to the respective law, GMOs can only be received by entities that hold a complete copy of the permit issued by the local regulatory authority responsible for overseeing work with genetically engineered organisms.

### 2.4 Legal implications associated with shipping of kit components

Shipping and/or exporting a kit also involves national and/or international regulations at various levels, such as customs and dual-use regulations, safety regulations and others. The following sections provide an overview of these regulations.

#### 2.4.1 Customs and dual-use regulations

When shipping material internationally, it is necessary to become familiar with customs regulation to prepare proper documentation that will be accepted by the carrier and the customs authorities in the destination country. Customs tariff numbers are harmonized internationally through the harmonized system (HS) by the world customs organization (WCO, www.wcoomd.org) providing a 6-digit number for each category of good. On the level of the European Union the 6-digit code is extended by further digits to assign various EU-specific customs regulations (digit 7 and 8). A ninth and 10th digit codes for EU-internal (TARIC, https://ec.europa.eu/taxation_customs) classifications and the 11th digit includes national regulations for each country. Customs declarations for outgoing goods have to specify the HS code, that can be looked up in the harmonized system database (https://harmonizedsystem.wcoomdpublications.org) hosted by the WCO. In addition, especially when exporting chemical and/or biological material it must be ensured that there are no export control requirements for dual-use items as specified in the regulation EC No. 202/821 (European Parliament and of the Council, 2009). Custom tariff numbers (HS) for the individual ATT kit components are: (i) pAIX-c: HS code 29349900, refers to nucleic acids and their salts, (ii) *E. coli* DH5 alpha: HS code 30024900, refers to toxins, cultures and microorganisms, (iii) A*. tumefaciens*: HS code 30024900, refers to cultures of microorganisms, (iv) *N. benthamiana*: HS code 12092990, refers to unspecified plant seeds.

#### 2.4.2 Hazardous materials and GMOs

The declaration of hazardous materials is another point which must be taken into account in particular when handling chemicals and biological material. Chemicals must be specified if they are toxic, explosive, corrosive or flammable. Biological material has to be declared when infectious, or when genetically modified. It should be noted that the untransformed Agrobacterium strain GV3101:pMP90RK without an expression vector is also a GMO, since it carries the modified Ti helper plasmid, which is required for efficient T-DNA transfer in the absence of virulence factors. Certain packaging aides like dry ice or liquid nitrogen must also be declared accordingly. For the ATT kit the following classifications have to be considered: (i) *E. coli* DH5 alpha: GMO/S1 organism, (ii) *A. tumefaciens:* GMO/S1 organism, (iii) dry ice (for shipping bacterial cultures): UN 1845, IATA PI 904.

### 2.5 Nagoja Protocol

Plant seeds are genetic resources and their use and distribution has been regulated in the “Nagoya Protocol on Access to Genetic Resources and the Fair and Equitable Sharing of Benefits Arising from their Utilization to the Convention on Biological Diversity” since ratification in 2014 (Smith et al., 2017). According to the protocol, the exchange of genetic resources of any kind should always include documentation certifying that access to a particular genetic resource used for a research project or commercial product development has been approved by the country of origin. Nagoya Protocol compliance regulations vary from country to country and from law to law. In principle, however, the use of a genetic resource that does not originate from the country of use requires an internationally recognized certificate of compliance (IRCC) as an athorization for access to genetic resources issued by the authorities of the provider country to fulfill duty of care. In the EU according to Article 4(3) (a) of the EU ABS Regulation, duty of care can be demonstrated with reference to an IRCC. In practice it means that the user of genetic resources will only need to provide information in DECLARE (https://webgate.ec.europa.eu/declare). There are country specific differences regarding the implementation of retroactive provisions. While EU recognizes the publication date of the Nagoja Protocol 20 May 2014, other countries like for example Brazil do not accept a certain retroactive due date, but expands the due diligence requirements towards all genetic resources ever acquired from Brazil. Anyhow, when using genetic resources from Brazil that were imported into the EU before May 2014, EU legislation will not penalize lack of documentation for these resources, still the user should be aware that this violates regulations in Brazil. In any case, when using a foreign genetic resource in the EU the origin of the material, as well the date and circumstances of its import should be well documented to ensure proper conduct in the context of Nagoja Protocol.

With respect to the genetic resources included in an ATT kit, no Nagoja Protocol issues are associated with the two modified bacterial strains *E. coli* and *A. tumefaciens*. The origin of *E. coli* DH5alpha cloning strain (Taylor et al., 1993) and *A. tumefaciens* GV3101is well documented and they have been in use worldwide for more than 20 years: In case of *N. benthamia* seeds, the situation is somewhat more difficult. Existing seeds in the scientific community have been imported long before 2014, but there is virtually no proper documentation on import procedures, dates and origin. As already mentioned in Section 2.5, the terms of export and commercial use of *N. benthamia* seeds from Australia under Nagoja regulations have not been fully clarified.

## 3 Conclusion

In theory, the idea of disseminating a comprehensive kit that allows academic (ATT kit for educational purposes) and corporate institutions (ATT kit for R&D) to get in touch with the possibilities of recombinant protein production through Agrobacterium-mediated transient transformation of plants seems straightforward, especially from the perspective of researchers who have been working regularly with the technology for several years. While it is a relatively easy exercise to summarize and structure the essential process steps, skills, materials and equipment associated with ATT-based protein expression (Section 1), the identification of legal and regulatory limitations and hurdles is complex and cumbersome. As the presented analysis shows, there are serious obstacles to both kit concepts (educational and R&D) that make implementation difficult at different levels. There are various regulations for the transport of chemicals, plant seeds, microorganisms, or even GMOs. As mentioned, adhering to safety regulations and proper declaration to enable smooth customs procedures is essential, and we have provided the most relevant custom tariff numbers in Section 2.4. For the transport of GMOs, an authorization to handle organisms of the appropriate biosafety level (BSL1) is also required from the receiving entity (Section 2.3).

Initially we assumed that it will be possible to obtain a commercial license for the dissemination of the *A. tumefaciens* strain GV3101:pMP90RK (Koncz and Schell, 1986). This strain is mandatory since it provides compatibility with the binary expression vector pAIX-c (a derivative of pTRA (Maclean et al., 2007)), that features FTO regarding all the included genetic elements (Section 2), which is another essential prerequisite for its commercial dissemination. Unfortunately, it is not possible to obtain a commercial license for GV3101:pMP90RK according to the German strain repository DSMZ and the regulations for the academic use of the strain also prevent the dissemination of the strain to third parties. As a result, GV3101:pMP90RK, one of the key components of a pAIX-c-based ATT kit cannot be included in a commercial ATT kit as originally planned. The second hurdle that was identified relates to the preferred plant species used for ATT, the native Australian plant *N. benthamiana*. Even though *N. benthamiana* seeds circulate in the scientific community since more than 2 decades, there is no proper documentation that enables compliance with the Nagoja Protocol, and a commercial license is currently not available due to ongoing regulatory issues in Australia. Although the binary vector pAIX-c can be used for commercial purposes, two other core elements of the ATT kit, the compatible disarmed *A. tumefaciens* strain GV3101:pMP90RK and *N. benthamiana* seeds, can neither be used commercially nor distributed to any third parties. Consequently, at this stage, only a minimal version of the ATT kit, containing the pAIX-c vector and the manual describing the agroinfiltration procedure, can be distributed to potential customers. The *A. tumefaciens* strain GV3101:pMP90RK and *N. benthamiana* plant seeds as well as the required chemicals and consumables have to be ordered individually by each user.

The described limitations regarding the free use of ATT components are probably the reason why this technology cannot yet be made available by commercial providers for research purposes or industrial applications. A completely different picture emerges for microbial or mammalian cell-based platforms used for production of recombinant proteins. Here, numerous systems, including possible licensing models, are available to produce proteins for research or industry. This has led to microbial cell systems such as bacteria or yeasts as well as mammalian and insect cell systems becoming standard in many laboratories and production facilities. In contrast, the plant ATT system is known almost exclusively to plant researchers and a handful of companies, even though this technology is uniquely capable of delivering even complex-folded, glycosylated proteins in a very short time. To enable the ATT technology to spread further, it is therefore imperative to establish ready-to-use kits and this in turn requires the availability of freely usable agrobacterial strains and *N. benthamiana* lines. The task is therefore to clearly clarify the origin and use of the organisms already present or to find alternative organisms. In the case of the plants to be infiltrated, a number of alternative plants, such as lettuce or spinach, are already being used today. However, the protein yield in these plants is significantly lower when compared to *N. benthamiana*, and their origin and usability must also be clarified.
